# Supplementary material for: ATAC-seq and MNase-seq Detect Distinct Modes of Chromatin Accessibility
Source: bioRxiv. 2026 Sep 11:2026.09.07.749956. Preprint. [Version 1] doi: 10.64898/2026.09.07.749956 (PMC13622623; doi:10.64898/2026.09.07.749956)
Supplement: Supplementary file 1 [file media-1.pdf]

Figure S1

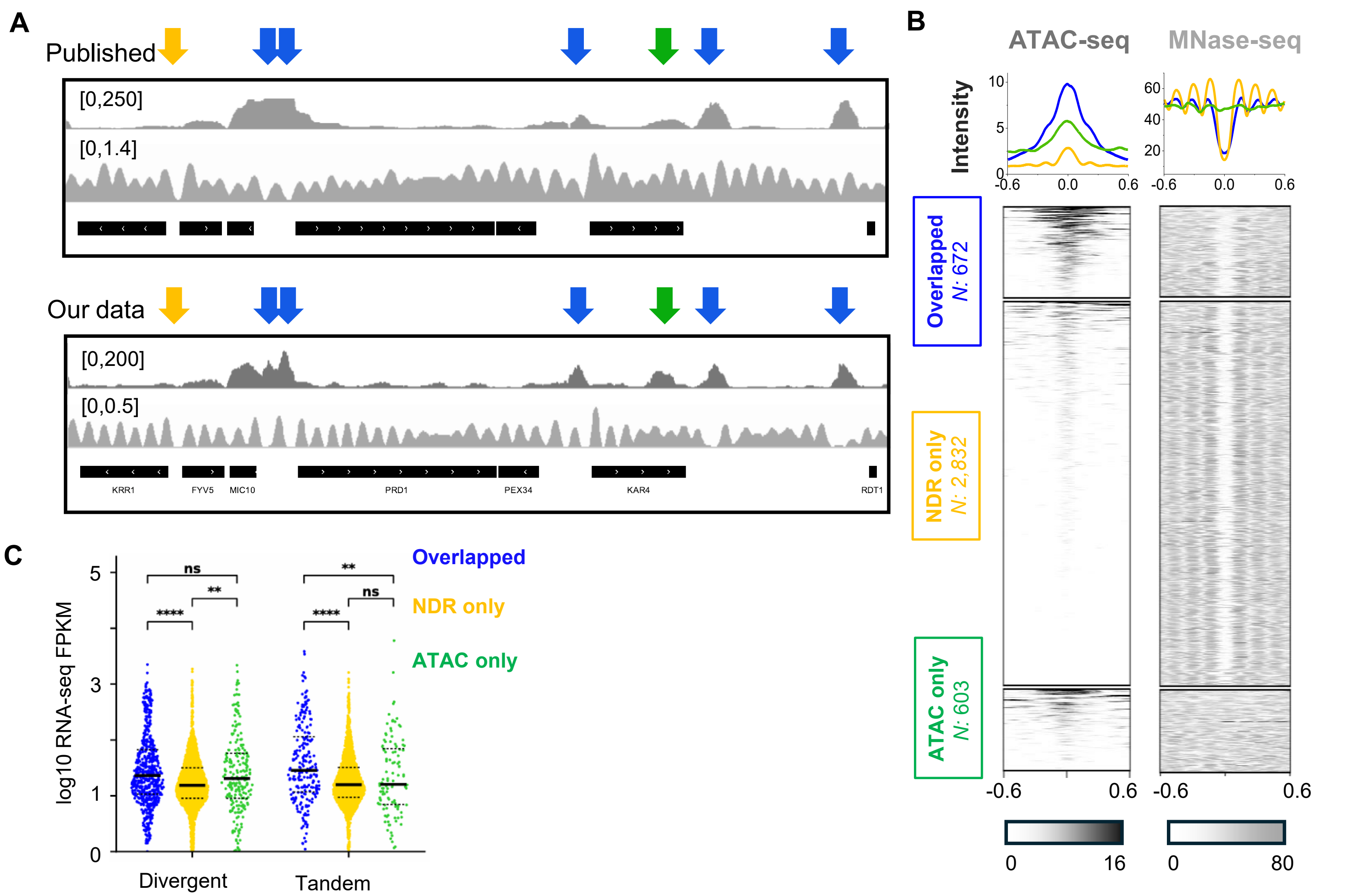

Figure S1. More data related to ATAC-seq peak and NDR compariosn. A) Additonal example of overlapped (blue), NDR-only (orange), and ATAC-only (green) regions. The top and bottom panels show the same genomic loci using published data[13,14] and our own data, respectively. **B)** Heat map of published dataset[13,14] using the same region coordinates in Figure 1A. **C)** Same analysis as in Figure 1F, performed using RNA-seq (steady-state RNA)[21] instead of RATE-seq (nascent RNA) data. ns: not significant, *P*-value < 0.01 (*), < 0.001 (**), <0.00001 (****).

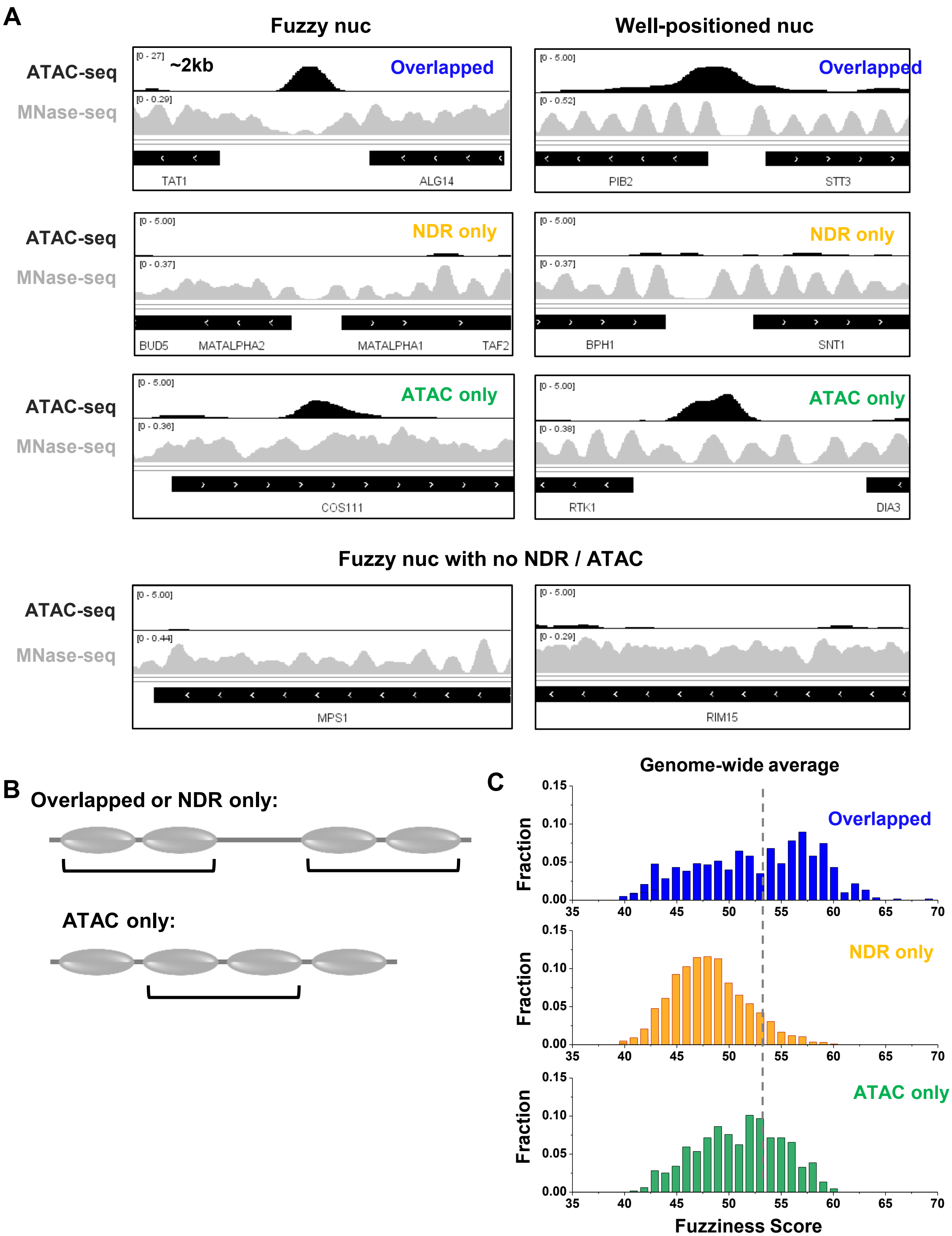

Figure S2. ATAC signals are not caused by "fuzzy" nucleosome positioning. A) Top three rows, examples of overlapped, NDR-only, and ATAC-only regions located near fuzzy (left) or well-positioned (right) nucleosomes. Bottom, examples of fuzzy nucleosome arrays that lack both ATAC-seq signal and NDRs. **B & C)** DANPOS analysis[22] of nucleosome fuzziness score. For overlapped and NDR-only regions, fuzziness scores were calculated for the four nucleosomes flanking each NDR, two on each side. For ATAC-only regions, fuzziness scores were calculated for the two nucleosomes at the center of each ATAC-seq peak. The distribution of fuzziness scores is shown in panel C.

Figure S3

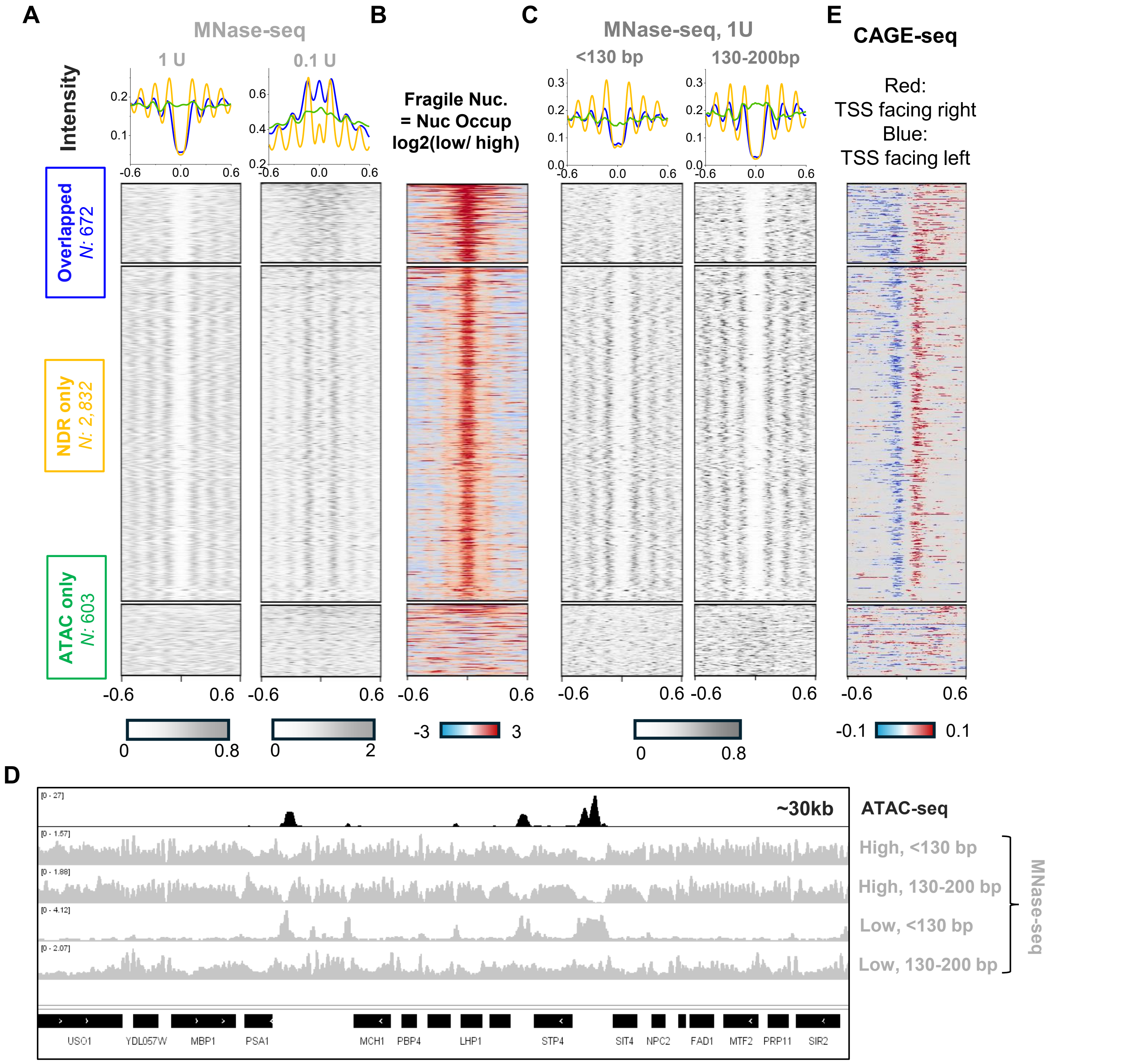

Figure S3. Additional MNase analysis. A) Aggregate MNase-seq profiles generated without fragment-size selection under high- and low-digestion conditions, using 1 U and 0.1 U MNase, respectively. **B)** Log2 fold difference between low- and high-MNase-seq signals. Higher values here are often used as an indicator of fragile nucleosomes. **C)** High-MNase-seq data separated by fragment size. Left: sub-nucleosomal (<130 bp), right: nucleosmsal (130-200 bp). **D)** Representitive genomic tracks of ATAC-seq and size-selected MNase-seq data generated under high- and low-MNase conditions. Note the similarity between ATAC-seq signal and the subnucleosomal fraction in the low-MNase data. **E)** CAGE-seq data[28] across overlapped, NDR-only, and ATAC-only regions.

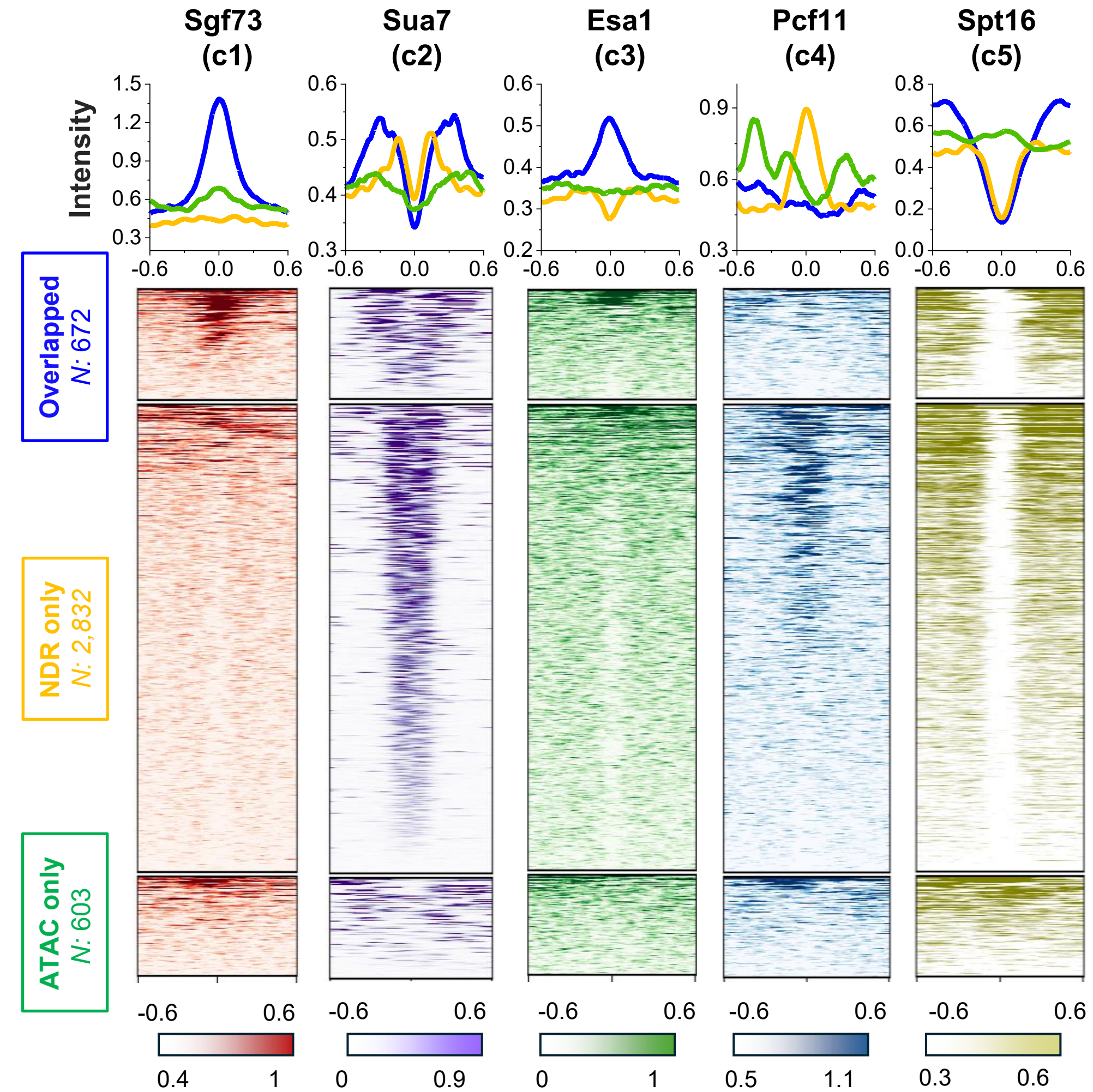

Figure S4. Examples of chromatin-associated factors from clusters c1–c5. The clusters of different binding patterns are defined in Figure 4B.

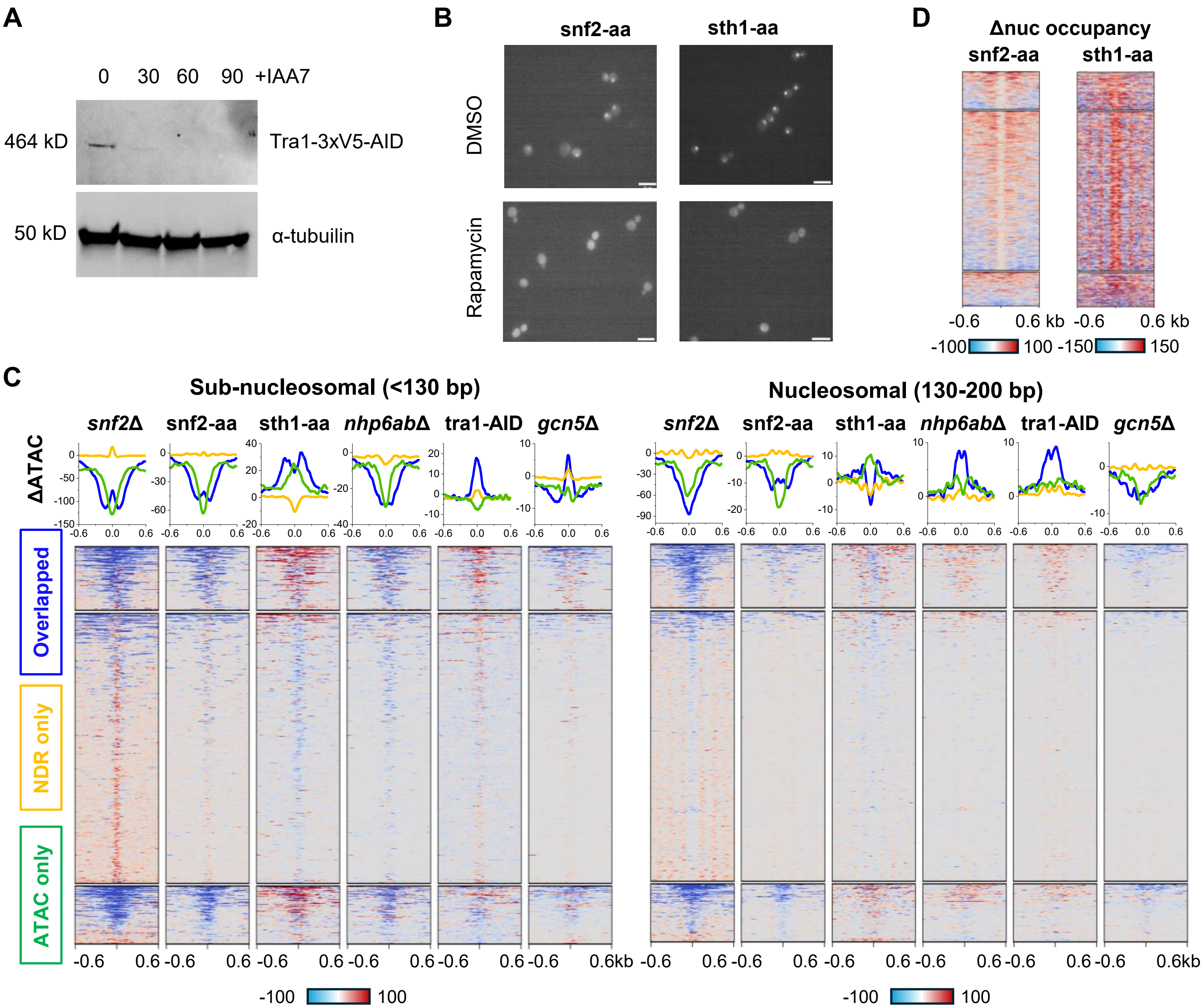

Figure S5. Supplementary data for perturbation experiment in Figure 5. A) Western blot confirming the auxin-induced degradation of Tra1. **B)** Imaging analysis confirming anchor-away depletion of Snf2 and Sth1. Both factors lose nuclear localization after rapamycin treatment. Scale bars are 10 µm. **C)** Changes in ATAC-seq signal following disruption of selected factors. The same data as in Figure 5B are shown, except that ATAC-seq reads here are separated by fragment size. **D)** Changes in nucleosome occupancy following Snf2 or Sth1 anchor-away. The Sth1 data are from our lab[40], and the Snf2 data are from literature[29].

Figure S6

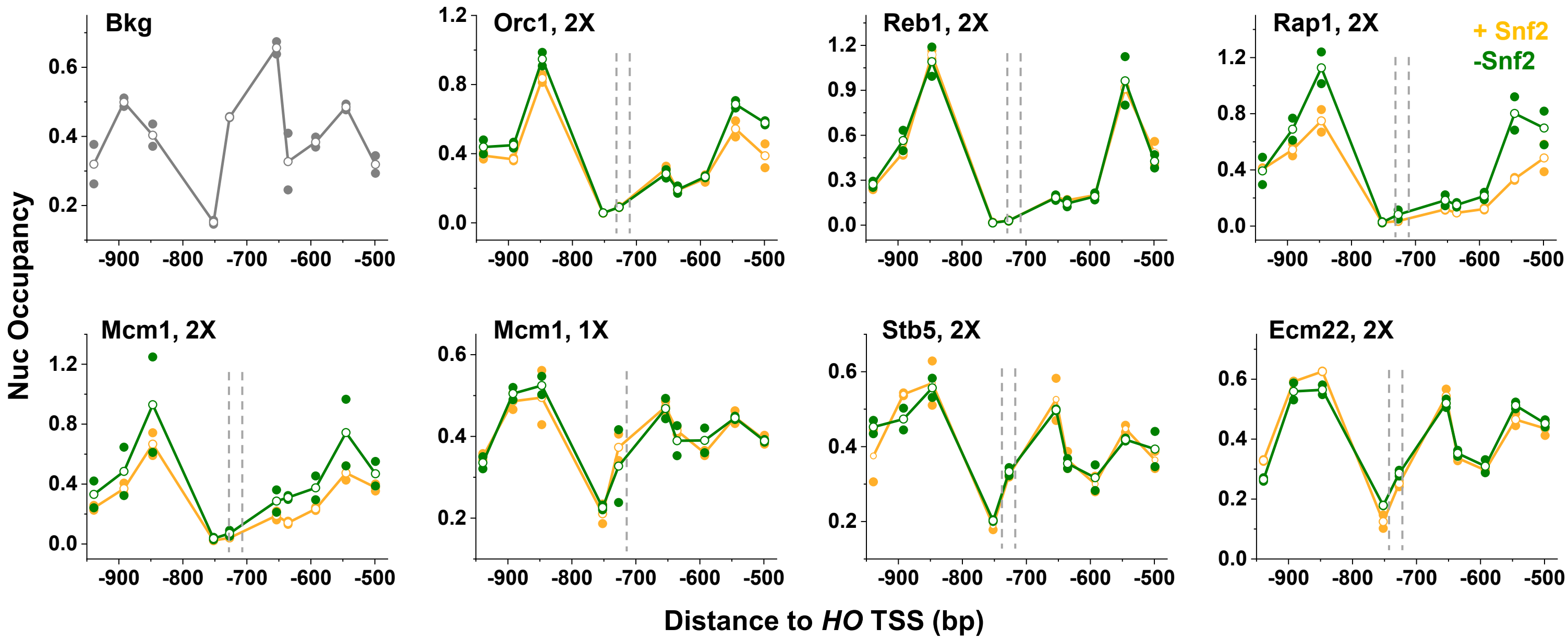

Figure S6. Tiling MNase-qPCR over *HOpr* containing different TF motifs. TF identities and motif copy numbers are indicated in each panel. The two curves show measurements before (orange) and after Snf2 anchor-away (green).
